# Potential risk sites and their relationship with dengue cases, Campinas municipality, Southeast Brazil

**DOI:** 10.1371/journal.pntd.0011237

**Published:** 2023-04-27

**Authors:** Jessica Andretta Mendes, Sophie O. Vanwambeke

**Affiliations:** 1 Department of Geography, Institute of Geosciences, University of Campinas, Campinas, Brazil; 2 Lancaster Medical School, Faculty of Health and Medicine, Lancaster University, Lancaster, United Kingdom; 3 Georges Lemaître Center for Earth and Climate Research, Earth & Life Institute, UCLouvain, Louvain-la-Neuve, Belgium; University of Queensland & CSIRO Biosecurity Flagship, AUSTRALIA

## Abstract

**Background:**

Among the main activities of dengue and vector control recommended by the Ministry of Health of Brazil is the inspection and monitoring of properties identified as Strategic Points (SPs) and Special Buildings (SBs). SPs are properties associated to hazard, where there is a concentration of suitable egg-laying containers for *Aedes aegypti* mosquitoes, while SBs have greater importance for human exposure to the dengue virus.

**Objectives:**

To investigate the effect of characteristics of the urban landscape on dengue incidence. Specifically, we tested if SPs and SBs affect dengue case distribution in Campinas, and if they do, if they affect the risk differently. We considered the period from 2013 to 2016.

**Methods:**

We tested whether dengue cases were more numerous than expected in the vicinity of SPs and SBs, putative sources of risk, using the Negative Binomial models. We also tested the existence of a gradient in incidence with increasing distance to SPs and SBs by using Stone’s test.

**Results:**

The Rate Ratios (RR) values were always higher closer to the SPs and SBs, and these values tended to decrease as distance from these sources increased. In general, RR values greater than one, which indicates a higher risk, were associated to the closest buffers from the SPs/SBs properties, until nearly 550 meters for the SPs and 650 meters for the SBs. Stone’s test results indicated that for all years considered, there was a correlation between the distance from the SPs/SBs and dengue cases occurrences, except for SBs from 2016. For SPs the relationship is stronger than for SBs.

**Discussion:**

Results are coherent with other studies which found that these properties contribute to an increased risk of dengue transmission. We emphasize the importance of public agents’ survey work and the importance to keep and improve the inspections in SPs/SBs recorded in Campinas.

## Author summary

Many factors drive dengue circulation and seasonal patterns, such as environmental factors, including the urban landscape, which can provide suitable sites for the emergence and proliferation of *Aedes aegypti*. We aim to test if some places of interest identified by the Ministry of Health in Brazil affect dengue case distribution and the risk differently. We considered the Strategic Points (SPs) and Special Buildings (SBs) as reported by public health authorities. These are properties that can favor the proliferation of mosquitoes and favor human exposure to the dengue virus. We focused on the municipality of Campinas from 2013 to 2016. We assessed the distribution of dengue cases around these properties and the existence of a gradient in risk with increasing distance from these places using Stone’s test. We found a correlation between the distance from these properties and dengue cases occurrences (for most sources investigated). Higher risk was always closer to the SPs and SBs, and the risk decreased as distance from these sources increased. Therefore, we emphasize the importance of public agents’ survey work and the maintenance and improvement of the inspections already conducted in SPs/SBs properties.

## Introduction

Dengue virus is transmitted by female mosquitoes mainly of the species *Aedes aegypti* and, to a lesser extent, *Aedes albopictus*. It is a member of the *Flaviviridae* family and there are four distinct, but closely related, serotypes of the virus (DENV-1, DENV-2, DENV-3, and DENV-4). Dengue virus may cause a severe, flu-like illness that affects infants, young children, and adults, but seldom causes death. Symptoms may include muscle and joint pains, nausea, vomiting, swollen glands, and a rash. Among the warning signs are severe abdominal pain, persistent vomiting, rapid breathing, bleeding gums, fatigue, restlessness, and blood in vomit. Recovery from infection with a specific serotype is believed to provide lifelong immunity against that serotype but severe disease has been associated with cross-immune reaction in the case of successive infection with different serotypes [1].

According to the World Health Organization [1], dengue is found in tropical and sub-tropical climates worldwide, mostly in urban and semi-urban areas. Tropical countries are most affected because of their environmental, climatic, and social conditions, particularly where proliferation of *Ae*. *aegypti* mosquitoes is continuous [2].

Many factors drive dengue circulation and seasonal patterns, such as the introduction of a new serotype in susceptible, dense populations, in places with high rates of infestation by *Ae*. *aegypti*. Cycles of low endemicity could be due, in part, to the decrease in population susceptible to the circulating serotype. Therefore, efforts should be applied continuously to reduce vector infestation.

*Ae*. *aegypti* mostly use artificial inappropriately discarded water containers to conduct immature development, such as cans, bottles, plastics, and used tires. Avoiding potential water reservoirs in backyards, the cleaning of unused/uninhabited lots, maintaining pools with treated water, and changing the water of aquatic plants periodically, including by the general population, is thus strongly recommended [3]. However, *Ae*. *aegypti* are adapting constantly and some studies report new breeding sites, such as raw sewage [4].

Studies conducted by de Mendonça et al. [5], Batista, Pinheiro; Santos Neto [6], and SUCEN [7] found that common *Ae*. *aegypti* breeding sites found in Brazil were flowerpots, and their plates and metallic containers (cans, pots, barrel).

Among the main activities of dengue and vector control, the Ministry of Health of Brazil recommends the inspection and monitoring of properties known they identify as Strategic Points (SPs) and Special Buildings (SBs) [8,9].

Inspectors identify SPs properties associated to hazard, where there is a concentration of suitable containers for breeding sites of *Ae*. *aegypti* mosquitoes. These properties can favor the proliferation of mosquito *larvae*, and significantly contribute locally to mosquito abundance (for example, in recycling material deposits, car and bus garages, construction sites, and cemeteries). Typically found in this category are commercial activities, using, storing or transporting egg-laying containers (such as tire repair shops, bottle deposits, container deposits, warehouses, and flower shops). Other properties, such as bus stations or airports, usually have a smaller number of suitable containers but contribute to passive dispersion of *Aedes aegypti*, with transport of goods and passengers [10].

SBs are monitored for their importance for human exposure to the dengue virus, due to the intense flow or presence of people. In addition, these properties can favor the proliferation of mosquitoes due to building complexity. Some examples of SBs are hospitals, military buildings, penitentiaries, hotels, religious temples, theaters, shopping malls, hypermarkets, zoos, clubs, parks, large industries, and teaching establishments [10].

Because the surveillance and vector control activities implemented in SBs generally require more effort and are more complex than in other properties, when a building is recorded as a Special Building, the surveillance team will work on it in a specific activity.

Therefore, considering the importance of SPs and SBs in the national and regional surveillance activities, we aimed to investigate the role of these putative sources of risk on dengue incidence. Specifically, we will test if SPs and SBs affect dengue case distribution in Campinas, and if they do, if SPs and SBs affect the risk differently.

Previous studies in Brazil found an association between the proximity of these sites (particularly the SPs) and increased risk (see Malavasi [11], Barbosa et al. [12], Johansen; do Carmo [13], and Mendes [14]), but others did not find such an association (Barbosa et al. [15] and Santos et al. [16]). Wijayanti, Octaviana and Nurlaela [17] pointed out that each ecological and socio-cultural setting has its own unique set of key containers, maybe explaining the inconsistency of results. Many landscape features are also associated to mosquito and dengue dissemination. Our assessment of the distribution of cases around SPs and SBs in Campinas will further assess the evidence generally and support the surveillance and control work locally.

## Material and methods

In short, we compared expected and observed dengue cases in the vicinity of SPs and SBs by using Poisson and the Negative Binomial regression models [18]. We compared both regression models by testing for overdispersion and performing the Likelihood Ratio test. We confirmed that the Negative Binomial regression was the best method to apply in the entire dataset.

In addition, we also tested the existence of a gradient in incidence with increasing distance to SPs and SBs by estimating the Rate Ratio (RR) and by using Stone’s test.

These procedures are described later in detail, especially during the section “Association between dengue cases and putative sources of risk”. All spatial data treatment was conducted in QGIS 3.4.11.

### Ethical approval

All data used is produced by public institutions and publicly accessible. No ethical approval of concerned patients was needed. The data used were only analyzed and presented in an aggregated form, the census grid cells. According to Resolution Number 510, of 07/04/2016, this type of research does not require an evaluation by the Research Ethics Committees and the National Research Ethics Committee (CEP/CONEP) [19].

### Study area description

**Fig 1:**
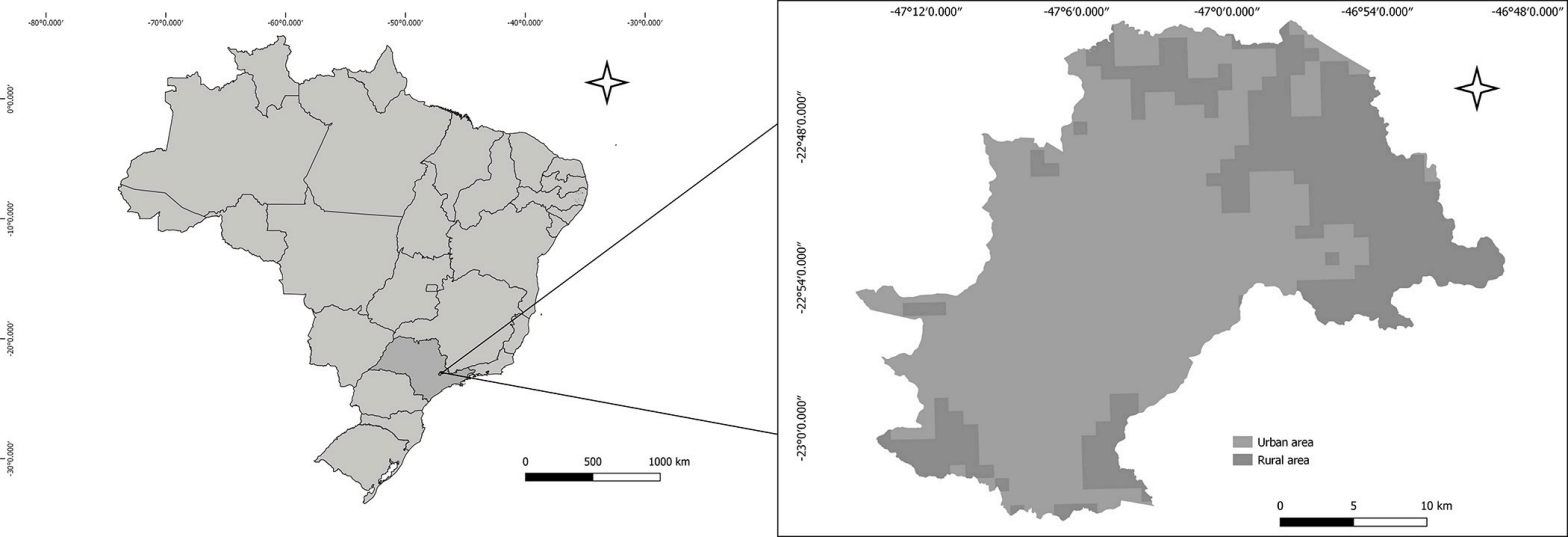
Campinas municipality. Source of base layer maps (federal units and municipality boundaries): https://www.ibge.gov.br/geociencias/organizacao-do-territorio/malhas-territoriais.html; source of base layer maps (census grids): ftp://geoftp.ibge.gov.br/recortes_para_fins_estatisticos/grade_estatistica/censo_2010/.

**Fig 2:**
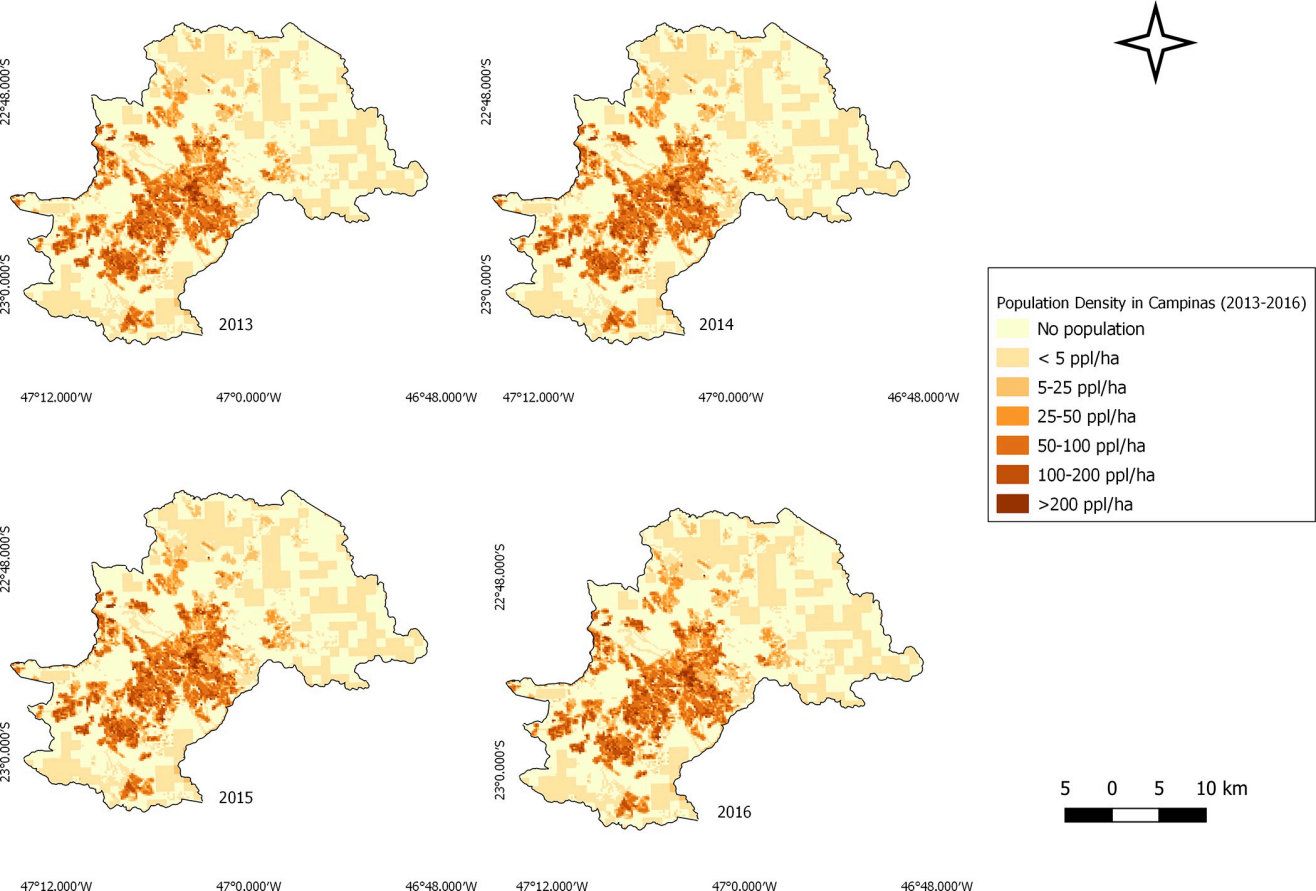
Campinas population density maps (2013–2016). Source of base layer maps (municipality boundary): https://www.ibge.gov.br/geociencias/organizacao-do-territorio/malhas-territoriais.html; source of base layer maps (census grids): ftp://geoftp.ibge.gov.br/recortes_para_fins_estatisticos/grade_estatistica/censo_2010/.

Our study area is the municipality of Campinas, located in São Paulo state (Figs 1 and 2). As of 2020, there were 1 213 792 inhabitants [20], 98% of whom live in urban areas [21]. The total area of the municipality is 794.57km^2^, and about 540km^2^ are considered urban.

The first autochthonous dengue cases in Campinas were registered in 1996. The area affected has since expanded as well as the number of cases. The first outbreak in 1997–1998 was restricted to the east of the municipality. In 2001–2002, cases clustered in the south. By 2007, the risk of dengue was widespread, occurring in most of the municipality [11].

Five years between 2000 and 2020 had high numbers (i.e. >300/100 000) [22,23,24] of dengue cases and incidence rates in Campinas: 2007 (1 100.93/100 000); 2013 (609.33 /100 000); 2014 (3 647.01/100 000); 2015 (5 638.18/100 000) and 2019 (2 185.08/100 000) [25,26,20,27].

**Fig 3:**
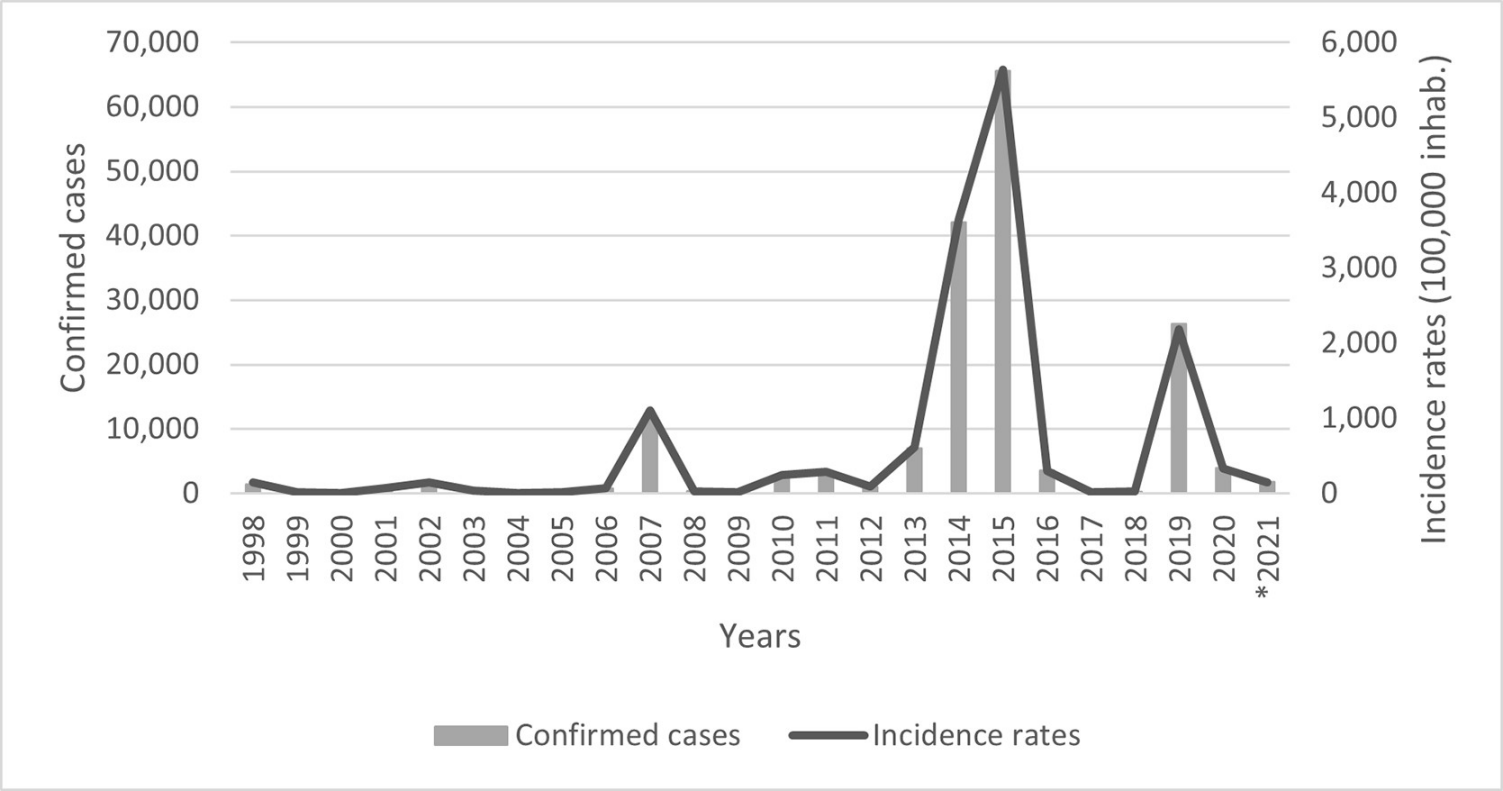
Dengue incidence rates and notified cases in Campinas, from 1998 to 2021. Source: Municipal Health Department [27] and IBGE [20,25,26]. *Dengue cases reported until June 15th, 2021.

We considered the period from 2013 to 2016, covering years with high and moderate number of dengue cases and incidence rates (Fig 3).

Conditions in Campinas favor *Ae*. *aegypti* infestations and circulation of dengue virus. High levels of precipitations in spring and summer (15 to 35 ml) and high temperatures (15 to 35°C) are typical [28]. Precipitations and temperatures increase around October and remain high until March. Precipitations and temperatures reach their lowest levels in June, July, and August.

Social issues have increased in the municipality since the 1980s, such as the emergence of suburbs with precarious infrastructure [29] which are favorable for *Ae*. *aegypti*. The south of the municipality was the most affected during the period 2007–2015 and this was associated to higher population densities and lower socioeconomic indicators, with inadequate housing and sanitation conditions [30]. Single family homes are predominant in Campinas, except in the center where there are mostly high-rise buildings. The north and east of Campinas are mainly middle- and high-income residential neighborhoods, with condominiums and empty lots, but there are also slums, and irregular occupations in these areas.

In Campinas, dengue control efforts have been constant. Since 2007, the municipal health secretary restructured the Municipal Program for Dengue Control (*Programa Municipal de Controle da Dengue*) [11]. Professionals were hired and trained, and control procedures were reviewed and standardized. More recently, vector control has been the focus of dengue control policies, for example by running regular larval density surveys [30]. Commonly recorded breeding sites are plant pots, plates, buckets, cans, jars and plastics, disposable bottles, among others. In addition, buildings known as Strategic Points (SPs) and Special Buildings (SBs) are also part of the entomological control activities (see “Putative sources of risk” below). Much information is provided for citizens on the Municipal Program for Dengue Control website [31]. The website lists information on vector control in houses, apartments and public spaces, epidemiological bulletins, dengue vaccine (Dengvaxia), and others. In 2017, a committee composed by representatives from a set of municipal entities was created to prevent and control arboviral diseases in the municipality (*Comitê Municipal de Prevenção e Controle das Arboviroses*).

## Population and Dengue data

We used population from the 2010 census, provided by the Brazilian Institute of Geography and Statistics (*Instituto Brasileiro de Geografia e Estatística–IBGE*), at the scale of census tracts and census grid cells. Census grid cells contain demographic data aggregated to cells of 1 000m resolution in rural areas, and 200m in urban areas. This is the most current population information available at this level of detail. The population in Campinas was 1 080 113 inhabitants in 2010. IBGE has estimated the population increase at municipality level. We estimated the population for the census grid cells using the population growth rate provided for the municipality by IBGE. The anonymized addresses of confirmed dengue cases were provided by the Department of Epidemiological Surveillance of Campinas. We considered the period between January 2013 and December 2016, totaling 118 658 confirmed dengue cases. Dengue case records were aggregated at the level of census grid cells.

## Putative sources of risk

Strategic Points and Special Buildings are recognized by the Brazilian Health Ministry as sites that constitute a potential risk for dengue transmission and that such properties should receive particular attention during surveillance activities. Inspection, larval search and vector control are carried out both in SPs and SBs. Because they are places vulnerable to heavy vector infestation, they must have a different routine for surveying and surveillance than other properties [32]. The frequency of visit varies according to the property’s field of activity and a score attributed during inspection. For example, in municipalities where we have *Aedes* mosquito infestation (house index > 0%), surveillance activities must take place every fortnight or monthly in SPs, and every three to six months in SBs.

Records of SPs or SBs must be continually updated and revised. Owners of sites registered as a SPs or SBs must implement prevention measures against mosquitoes and dengue until an annual update of registries can remove them from the SPs or SBs records. The number of properties registered as SPs or SBs, as well as their location thus varies from year to year [10].

In the State of São Paulo, sites classified as SPs or SBs are recorded in the public database of the Superintendence for the Control of Endemic Diseases of the State (SUCEN). We used records of SPs and SBs for the municipality of Campinas from 2013 to 2016. We found 1 089 records, 882 SPs and 207 SBs, detailed as follows: 161 SPs in 2013; 225 in 2014; 257 in 2015; and 239 in 2016; 59 SBs in 2013; 46 in 2014; 51 in 2015; and 51 in 2016. No further selection, e.g. based on field of activity, was made. We considered all sub-categories recorded by the surveillance authorities. Each record has an identification number, address, the year in which it was registered, the activity of the property, the number of receptacles/breeding sites inspected during the surveillance work, and the number of *Ae*. *aegypti* and/or *Ae*. *albopictus* larvae and adult mosquitoes found.

## Dengue cases per census grid cells

Dengue cases were aggregated and summed to census grid cells. Then, for each cell of the grid, we created a centroid, associated to the number of cases and updated population. This avoids an overestimation of the number of cases by double counting when buffers around SPs and SBs are overlaid on cells.

## Distance and proximity areas

**Fig 4:**
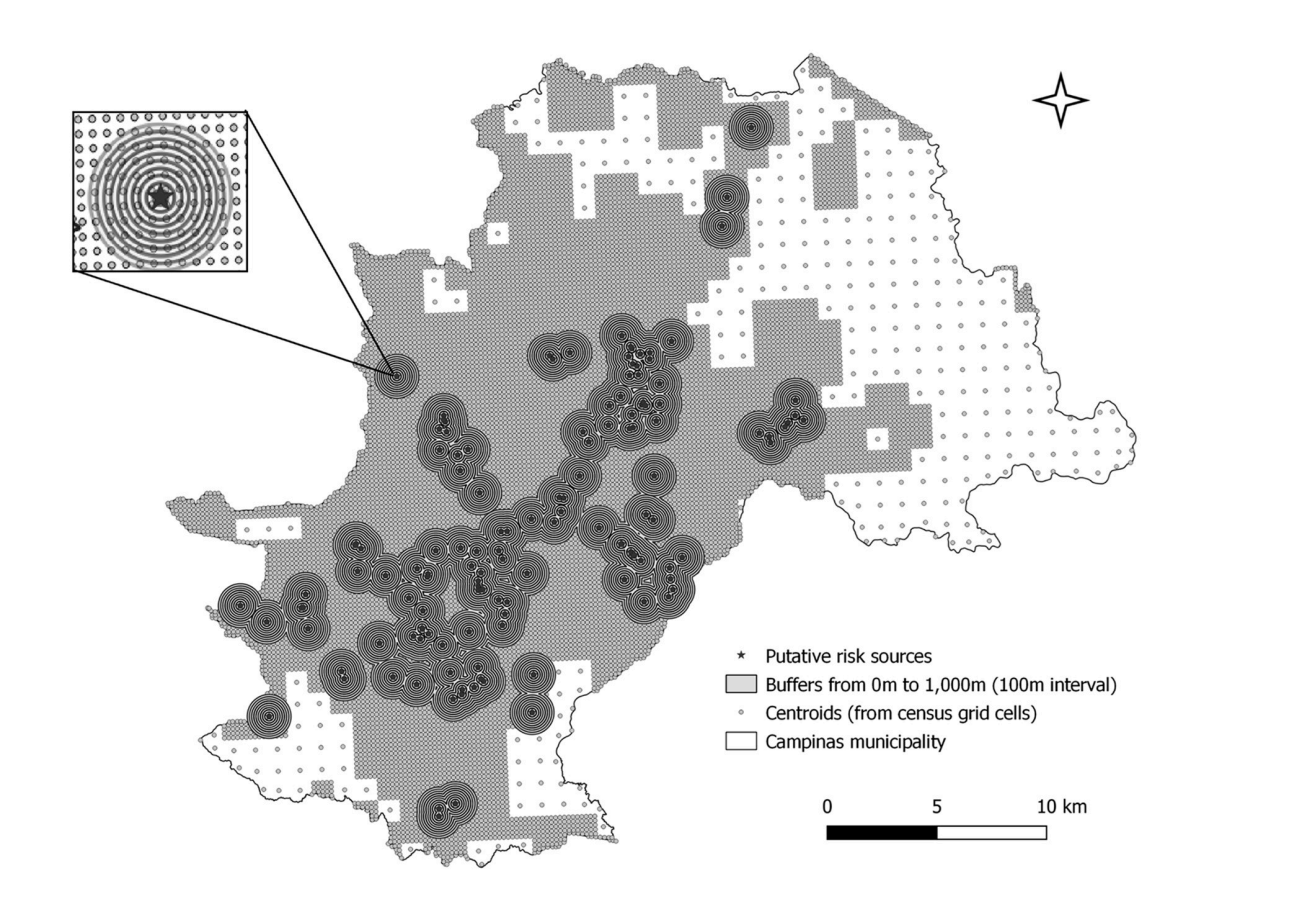
Buffers from putative risk sources and their intersection to the centroids of census grid cells. Source of base layer map (municipality boundary): https://www.ibge.gov.br/geociencias/organizacao-do-territorio/malhas-territoriais.html; source of base layer map (census grids): ftp://geoftp.ibge.gov.br/recortes_para_fins_estatisticos/grade_estatistica/censo_2010/.

Buffers were created around SPs and SBs at increasing distances, by intervals of 100m, up to 1 000m from the sources. We considered previous studies that argue that the main dengue vector in Brazil, *Ae*. *aegypti* can fly up to 800m [33, 34]. We then calculated the number of dengue cases and estimated the population for each buffer, using the intersection with grid cells centroids (Fig 4).

## Association between dengue cases and putative sources of risk

The data were separated by year (2013 to 2016) and by group (analyses for SPs and for SBs). We used the R statistical software (Version 4.0.3) to apply the Log-linear Poisson Regression model and the Negative Binomial Regression, to calculate the rate ratios (RR) values and to run the Stone’s test. Detailed information about the data, packages, and the functions (including the script) can be found in S1 and S2 Files).

The response variable considered was the number of dengue cases in each distance range (buffer), a count variable, while the explanatory variable is the mean distance for each buffer range (50m; 150m; 250m and so on). In our first attempt, we applied a Log- linear Poisson Model, using the function *glm* in R, including the population in the offset component, defined as follows [35]:

$$\log\left(E(Y)\right)=\beta_{0}+\beta_{1}x_{i}+log(t_{i})$$ (1)

Next, we tested for overdispersion using the package *AER* and the function *dispersiontest*. The overdispersion is the condition whereby the variance is higher than the mean. We found *p-values* <0.05 for the data on Strategic Points (SPs) from 2014, 2015 and 2016, indicating overdispersion. We thus applied the Negative Binomial Regression Model using the package MASS and the function *glm*.*nb*. The Negative Binomial distribution is a form of the Poisson distribution in which the distribution’s parameter is itself considered a random variable. The variation of this parameter can account for data variance higher or lower than the mean [18]:

$$\mu_{i}=exp(X_{i}\beta_{i}+e_{i})=\exp\left(X_{i}\beta_{i}\right)exp(e_{i})$$ (2)

Where *exp*(*e*_*i*_)~ *Gamma* (*α*^−1^, *α*^−1^)

As the population size in each distance range is different, we included an offset variable [36] to control for population at risk in each of the distance ranges.

The RR values were calculated using the unconditional maximum likelihood estimation (Wald), using the package *epitools* and the function *rateratio*.*wald*. Values higher than one indicated an increased risk compared to the overall incidence. To estimate the risk, the observed number of cases must be compared to an expected number of cases, which is implicit in the regression model. Here, we used a separate computation to estimate the expected number of cases.

To compute the expected number of cases for each range of distance, we applied the indirect standardization method [35, 37, 38, 39], in which the expected number of cases in each area is calculated according to [35]:

$$E_{i}=P_{i}*r^{+}$$ (3)

Where, *P*_*i*_ is the total population in area *i* and *r*^+^ is the overall incidence ratio, at the scale of the largest buffer. This means that if there are further sources of spatial heterogeneities at the scale of the city or population they will not interfere with the estimation of expected number of cases.

$$r^{+}=\frac{O^{+}}{P^{+}}$$ (4)

Where, *O*^+^ is the sum of number of cases in each buffer range and *P*^+^ is the sum of population in each buffer range.

In ecological studies, including studies using Stone’s test, data are compared at the population-level, for example, as applied by Shaddick & Elliott [38], Masseret et al. [39], Demoury et al. [40] and Stone [41]. We thus used a constant value for incidence for the total buffered area to estimate the expected number of cases.

The expected number of cases were used for both the RR estimation and also to the Stone’s test. The latest is used to assess risk around putative risk sources [38] and we used it in order to test the role of SPs and SBs as sources of infection. For the Stone’s test we used the packages *spdep* and *DCluster* and the function *stone*.*test*. If we consider the ordered relative risks of the regions according to their distances to the source, the test is as follows [35]:

$$Max\;i\frac{\sum_{j_{=1}}^{i}O_{j}}{\sum_{j_{=1}}^{i}E_{j}}$$ (5)

The null hypothesis is that relative risks are constant across space, while the alternative is that there is a decreasing trend in relative risk as distance to the putative source increases [42]. In Stone’s test, subregions (buffer ranges) are ordered by distance away from the putative source [43]. For each subregion, the ratio of the cumulative number of observed cases to the cumulative number of expected cases is computed. Stone’s test statistic is the maximum of these ratios. Its significance is evaluated by comparing it to a simulation of the null hypothesis.

## Results

### Descriptive analyses

Over the period 2013–2016, 118 658 confirmed cases of dengue were diagnosed in Campinas. 104 439 cases were included in the analyses. Discarded cases had inconsistent, incomplete, or absent location data.

**Fig 5:**
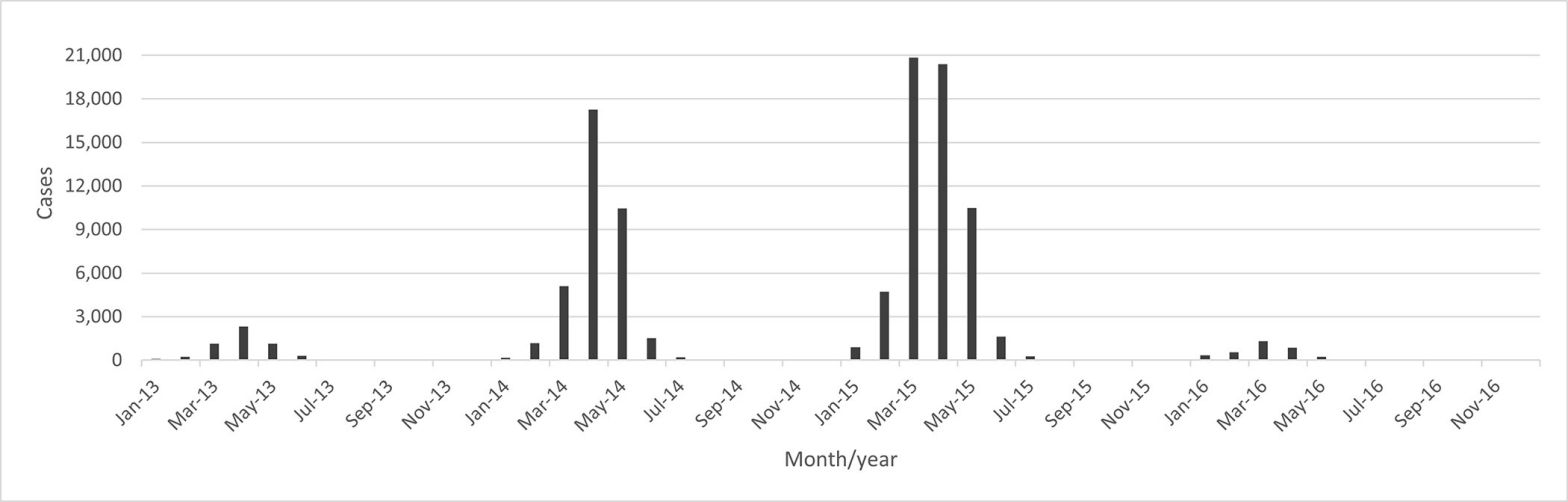
Distribution of dengue cases from 2013 to 2016. Source: produced based on data provided by the Health Department of Campinas.

Most cases occurred in 2014 and 2015. Seasonality was marked, with higher incidence in March, April, and May (Fig 5).

### Dengue cases around putative sources

Tables 1 and 2 show the number of cases observed, the population and the number of cases expected estimated by buffer distance, for each year of study and for each category of putative risk source (SPs and SBs).

**Table 1:** Observed dengue cases and the estimation of population and expected dengue cases in each buffer from Strategic Points (2013–2016).

Strategic Points (SPs)												
	2013			2014			2015			2016		
Buffers (meters)	Observed	Expected	Population	Observed	Expected	Population	Observed	Expected	Population	Observed	Expected	Population
50	197	109.99	28 644	1 596	858.21	29 015	2 535	1 742.78	38 271	131	81.91	26 991
150	365	242.86	63 247	3 880	2 364.20	79 931	5 154	3 658.07	80 330	268	181.30	59 745
250	407	290.96	75 775	3 984	2 725.88	92 159	7 308	5 633.37	123 707	364	240.81	79 354
350	558	450.55	117 338	3 739	3 108.06	105 080	6 865	5 742.16	126 096	348	266.99	87 983
450	434	384.04	100 015	3 148	3 171.86	107 237	6 681	6 182.83	135 773	323	271.52	89 474
550	421	418.21	108 916	2 939	3 362.70	113 689	5 158	5 282.18	115 995	353	403.46	132 952
650	314	414.77	108 019	2 505	3 447.82	116 567	4 537	5 263.83	115 592	273	358.66	118 190
750	292	386.96	100 777	2 427	3 468.79	117 276	3 669	4 995.34	109 696	195	282.93	93 234
850	284	419.30	109 198	1 932	2 689.53	90 930	2 740	4 379.48	96 172	203	298.95	98 512
950	234	388.36	101 142	1 891	2 843.96	96 151	2 085	3 851.97	84 588	150	221.48	72 983
Total	3 506 (O+)*	3 506.00	913 071(P+)*	28 041(O+)*	28 041.00	948 035(P+)*	46 732(O+)*	46 732.00	1 026 220(P+)*	2 608(O+)*	2 608.00	859 418(P+)*

*Note: O+ is the sum of number of cases in each buffer range and P+ is the sum of population in each buffer range.

**Table 2:** Observed dengue cases and the estimation of population and expected dengue cases in each buffer from Special Buildings (2013–2016).

Special Buildings (SBs)												
	2013			2014			2015			2016		
Buffers (meters)	Observed	Expected	Population	Observed	Expected	Population	Observed	Expected	Population	Observed	Expected	Population
50	27	17.61	5 142	206	173.80	5 776	396	351.48	8 618	26	24.26	7 686
150	115	75.69	22 099	566	431.77	14 349	1 344	1 210.15	29 672	73	62.03	19 653
250	171	133.08	38 855	1 052	918.55	30 526	1 933	1 863.48	45 691	193	177.85	56 351
350	194	151.87	44 343	1 386	1 178.08	39 151	1 913	1 716.04	42 076	139	115.99	36 753
450	214	179.58	52 433	1 482	1 383.99	45 994	2 813	2 608.12	63 949	205	194.06	61 490
550	240	236.27	68 986	1 353	1 267.63	42 127	2 431	2 306.48	56 553	172	191.53	60 687
650	232	243.81	71 186	1 346	1 316.26	43 743	2 684	2 722.72	66 759	129	127.59	40 429
750	255	290.04	84 684	1 428	1 577.29	52 418	2 761	2 889.82	70 856	198	200.41	63 502
850	206	255.48	74 594	1 285	1 481.78	49 244	3 061	3 367.04	82 557	167	166.13	52 638
950	277	347.57	101 482	1 446	1 820.85	60 512	2 920	3 220.66	78 968	175	217.15	68 805
Total	1 931(O+)*	1 931.00	563 804(P+)*	11 550(O+)*	11 550.00	383 840(P+)*	22 256(O+)*	22 256.00	545 699(P+)*	1 477(O+)*	1 477.00	467 994(P+)*

*Note: O+ is the sum of number of cases in each buffer range and P+ is the sum of population in each buffer range.

We identified that the number of cases observed was always higher than expected closer to SPs (from 0 to 100m). In 2013, we observed this pattern until 500 – 600m; in 2014 until 300 - 400m; in 2015 until 400 – 500m; and in 2016 the observed number of cases was higher than expected until 400 – 500m. Further than that, the number of observed cases was lower than the number of expected dengue cases. The number of observed dengue cases was always lower than the observed beyond 900m (Table 1).

Closer to SBs (from 0 to 100m), the number of cases was always higher than expected. In 2013, we observed the same pattern until 500 – 600m; in 2014 until 600 - 700m; in 2015 until 500 - 600m; and in 2016 until 400 – 500m. Further, the number of observed cases was lower than the number of expected dengue cases, except for 2016. For SBs as well, the number of observed dengue cases was always lower than the expected beyond 900m (Table 2).

### Association between dengue cases and putative sources

The Negative Binomial Regression models results showed negative coefficients for both categories (SPs and SBs) for all the years analyzed with *p-values* <0.001, which confirms that the number of dengue cases are negatively associated with distance from the putative risk sources: as the distance increases, the number of cases decreases (Table 3)

**Table 3:** Summary of results for the Negative Binomial Regression models.

Summary of Results: Negative Binomial Regression (Strategic Points—SPs)								
Source	Coefficients	Estimate	Standard Error	z_value	Pr(>|z|)	Degrees of Freedom	Residual Deviance	AIC
SPs_2013	(Intercept)	-4.93	3.57*10^−2^	-138.17	<0.001	8	6.9244	89.47
	Distance Range	-1.22*10^−3^	6.58*10^−5^	-18.49	<0.001			
SPs_2014	(Intercept)	-2.89	5.45*10^−2^	-53.1	<0.001	8	10.057	143.12
	Distance Range	-1.23*10^−3^	9.48*10^−5^	-12.97	<0.001			
SPs_2015	(Intercept)	-2.56	3.36*10^−2^	-76.12	<0.001	8	10.207	143.13
	Distance Range	-1.12*10^−3^	5.89*10^−5^	-19.02	<0.001			
SPs_2016	(Intercept)	-5.18	5.85*10^−2^	-88.67	<0.001	8	9.8249	96.08
	Distance Range	-1.19*10^−3^	1.05*10^−4^	-11.33	<0.001			
Summary of Results: Negative Binomial Regression (Special Buildings—SBs)								
Source	Coefficients	Estimate	Standard Error	z_value	Pr(>|z|)	Degrees of Freedom	Residual Deviance	AIC
SBs_2013	(Intercept)	-5.18	5.81*10^−2^	-89.169	<0.001	8	2.3108	77.94
	Distance Range	-8.04*10^−4^	9.08*10^−5^	-8.862	<0.001			
SBs_2014	(Intercept)	-3.18	2.79*10^−2^	-114	<0.001	8	11.418	109.11
	Distance Range	-5.39*10^−4^	4.50*10^−5^	-12	<0.001			
SBs_2015	(Intercept)	-3.04	2.30*10^−2^	-132.145	<0.001	8	9.7108	117.06
	Distance Range	-2.67*10^−4^	3.71*10^−5^	-7.193	<0.001			
SBs_2016	(Intercept)	-5.56	6.33*10^−2^	-87.871	<0.001	8	7.9976	81.04
	Distance Range	-3.38*10^−4^	1.02*10^−4^	-3.319	<0.001			

**Fig 6:**
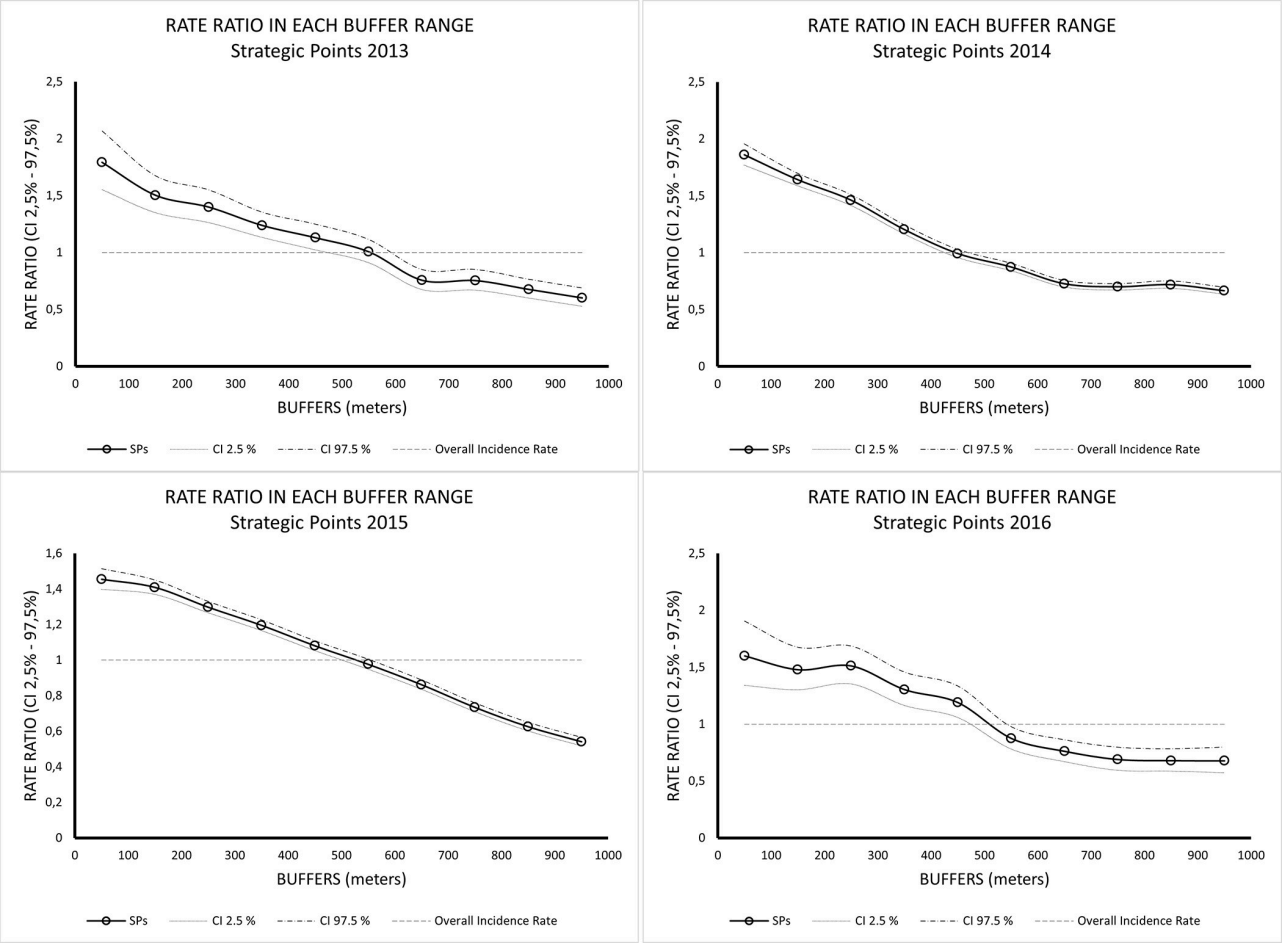
Rate Ratio (RR) values estimated based on observed and expected dengue cases for each buffer from the Strategic Points (2013–2016).

RR values were always higher closer to the SPs (0-100m) and these values tend to decrease as distance from these sources increases. In 2013, RR values higher than one were found in buffers from 0 to 550 meters from the SPs; in 2014, RR values higher than one were found in buffers from 0 –450m; in 2015 and 2016, RR values higher than one were found in buffers from 0 to 550 meters from the SPs (Fig 6 and Table 4).

**Table 4:** Rate Ratio (RR) values and their respective confidence interval for each buffer from the Strategic Points (2013–2016).

Strategic Points (SPs)																
	2013				2014				2015				2016			
Mean distance	RR	2.50%	97.50%	p-value	RR	2.50%	97.50%	p-value	RR	2.50%	97.50%	p-value	RR	2.50%	97.50%	p-value
50m	1.79112	1.55167	2.06753	<0.001	1.859693	1.768221	1.955896	<0.001	1.454569	1.397577	1.513885	<0.001	1.599371	1.341944	1.90618	<0.001
150m	1.50295	1.34937	1.67402	<0.001	1.641147	1.586965	1.697178	<0.001	1.408941	1.368987	1.45006	<0.001	1.478189	1.303552	1.676223	<0.001
250m	1.39882	1.26237	1.55001	<0.001	1.461546	1.413841	1.51086	<0.001	1.29727	1.265677	1.329651	<0.001	1.511574	1.354573	1.686772	<0.001
350m	1.23848	1.13264	1.3542	<0.001	1.203002	1.162644	1.244761	<0.001	1.195543	1.165637	1.226217	<0.001	1.303399	1.165465	1.457658	<0.001
450m	1.1301	1.02283	1.24862	<0.05	0.992478	0.956579	1.029724	0.688	1.080573	1.053224	1.108632	<0.001	1.189604	1.059725	1.335402	<0.01
550m	1.00666	0.90987	1.11375	0.898	0.874001	0.841412	0.907853	<0.001	0.976491	0.94881	1.004979	0.105	0.874937	0.782894	0.977801	<0.05
650m	0.75705	0.6745	0.8497	<0.001	0.726546	0.697449	0.756856	<0.001	0.86192	0.836047	0.888594	<0.001	0.761165	0.671943	0.862234	<0.001
750m	0.7546	0.66968	0.85028	<0.001	0.699667	0.671245	0.729293	<0.001	0.734485	0.710214	0.759586	<0.001	0.689219	0.595887	0.79717	<0.001
850m	0.67732	0.60018	0.76438	<0.001	0.718341	0.685977	0.752233	<0.001	0.625645	0.602	0.650219	<0.001	0.679053	0.58868	0.7833	<0.001
950m	0.60253	0.52784	0.68778	<0.001	0.664919	0.634666	0.696614	<0.001	0.541282	0.518049	0.565557	<0.001	0.677277	0.574507	0.798431	<0.001

**Fig 7:**
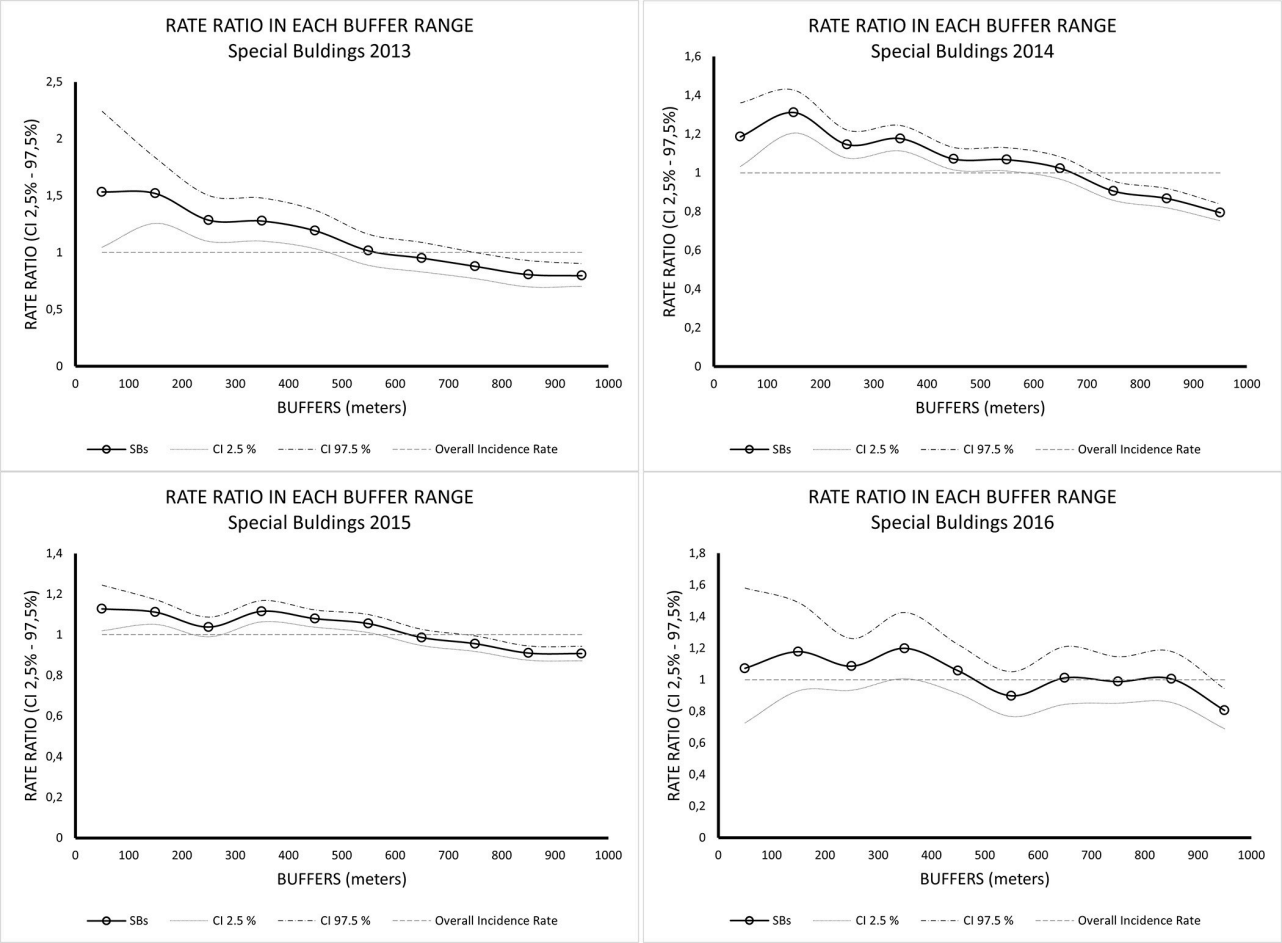
Rate Ratio (RR) values estimated based on observed and expected dengue cases for each buffer from the Special Buildings (2013–2016).

RR values for the SBs category (Fig 7) were also higher than one closer to the sources (0 – 100m). These values tend to decrease as distance from these sources increases. However, the decreasing trend as the distance increases is not as evident as for SPs. We noticed a clearer spatial variation in RR values. In 2013, RR values higher than one were found in buffers from 0 to 550 meters from the SB; in 2014, RR values higher than one were found in buffers from 0 –650m; in 2015, RR values higher than one were found in buffers from 0 to 650m. In 2016, there was a greater oscillation in RR values higher than one: from 0 to 550m, then, 650m, and 850m from the SBs (Table 5).

**Table 5:** Rate Ratio (RR) values and their respective confidence interval for each buffer from the Special Buildings (2013–2016).

Special Buildings (SBs)																
	2013				2014				2015				2016			
Mean distance	RR	2.50%	97.50%	p-value	RR	2.50%	97.50%	p-value	RR	2.50%	97.50%	p-value	RR	2.50%	97.50%	p-value
50m	1.53313	1.048631	2.24147	<0.05	1.185245	1.032704	1.360319	<0.05	1.126665	1.0200968	1.244366	<0.05	1.0718469	0.727337	1.579538	0.7258
150m	1.5194	1.258829	1.8339	<0.001	1.31088	1.204809	1.42629	<0.001	1.110602	1.0511127	1.173458	<0.001	1.1769386	0.930456	1.488715	0.1737
250m	1.28498	1.098958	1.50248	<0.01	1.145286	1.07523	1.219907	<0.001	1.037307	0.9902013	1.086653	0.122	1.0852142	0.934038	1.260859	0.2852
350m	1.27739	1.102081	1.48058	<0.01	1.176491	1.112735	1.2439	<0.001	1.114773	1.0639127	1.168065	<0.001	1.1983448	1.007078	1.425938	<0.05
450m	1.19167	1.034733	1.3724	<0.05	1.070817	1.014445	1.130322	<0.05	1.078554	1.0370721	1.121696	<0.001	1.0563532	0.912782	1.222507	0.4619
550m	1.01577	0.888253	1.1616	0.819	1.067347	1.008896	1.129183	<0.05	1.053987	1.0107715	1.099051	<0.05	0.8980336	0.766856	1.05165	0.1817
650m	0.95157	0.830411	1.0904	0.475	1.022598	0.966471	1.081983	0.438	0.985777	0.9470787	1.026057	0.483	1.0110124	0.844516	1.210333	0.905
750m	0.87919	0.77156	1.00184	0.0532	0.905349	0.856917	0.956518	<0.001	0.955423	0.9183769	0.993964	<0.05	0.9879559	0.851762	1.145927	0.8728
850m	0.80632	0.698425	0.93089	<0.05	0.867198	0.818628	0.918649	<0.001	0.909108	0.8753998	0.944114	<0.001	1.0052565	0.856613	1.179693	0.9488
950m	0.79696	0.702664	0.90391	<0.001	0.794136	0.751884	0.838763	<0.001	0.906646	0.8723365	0.942305	<0.001	0.8058938	0.689014	0.942601	<0.01

For SPs there is a correlation between exposure distance and increase in the number of disease events (Stone’s test > 1; *p-value* <0.05; Table 6). For SBs, we also found a correlation between exposure distance and increase in the number of disease events, except those related to 2016 (*p-value* >0.05).

**Table 6:** Stone’s Test statistics and their respective *p-values* for buffers from Strategic Points and Special Buildings (0–1 000m, 100m interval) recorded from 2013 to 2016.

	Strategic Points		Special Buildings	
	Stone’s Test Statistic	p-value	Stone’s Test Statistic	p-value*
2013	1.79	0.01	1.51	0.02
2014	1.86	0.01	1.27	0.01
2015	1.45	0.01	1.12	0.02
2016	1.60	0.01	1.15	0.24

*Note: p-values derived from Stone’s Test.

## Discussion

The highest number of cases occurred during 2014 and 2015. This may be related to dengue serotype circulation, population immunity or climatic conditions. As reported by Johansen, Carmo, and Alves [44], in 2007 the epidemic in Campinas was mainly caused by DENV-3 serotype and in 2014 by DENV-1 serotype. The high number of confirmed cases and high incidence rates in 2014 suggests a population that never had extensive contact with the DEN-1 serotype. The DENV-1 serotype continued circulate during 2015 and 2016 [45].

We also observed a seasonal pattern of confirmed dengue cases, occurring mainly in March, April, and May, coherently with the seasonal variation in temperature and rainfall known to influence vector dynamics and dengue incidence in Brazil. Böhm et al. [23] reported that studies performed in Brazil and Latin America between 2000 and 2012 pointed at cyclical transmission peaking during the rainy seasons.

Viana and Ignotti [46] found in their review that larval density and the number of dengue cases increased in four municipalities from the State of São Paulo during the first four months of the year, the period of high levels of rainfall and decreased between June and September, when rainfall is lower. In São Sebastião, State of São Paulo, the highest indices of *Ae*. *aegypti* larval density were observed from November to April, when the highest temperature and relative air humidity were registered. They mentioned the relevance of considering a time lag: in São Sebastião, São Paulo, the rain and temperature of a given month contributed to explain the number of cases of dengue two to four months later. While it is important to recognize that the factors related to dengue epidemics vary between location and time [2], a seasonal pattern that may relate to temperature and rainfall is visible in Campinas.

We observed that the RR values were always higher closer to the SPs and SBs, and these values decreased as distance from these sources increased. In general, RR values greater than one, which indicate a higher risk, were associated to the closest buffers from the SPs/SBs properties, until nearly 400–600 meters from these sources. Our Stone’s test results indicated that for all years considered, there was a correlation between the distance from the SPs/SBs and dengue cases occurrences, with statistics values higher than one and significant *p-values* (<0.05), except for SBs from 2016 (*p-value* = 0.26).

The fact that the population sometimes decreased with increasing distance from the SPs/SBs highlights the fact that those places identified as SPs and SBs are often found in the most densely populated parts of Campinas, or that increasing distances simply captures the less populated outskirts. Either way, it confirms the relevance of focused attention and control as implemented by the identification of SPs and SBs.

Poisson regression is commonly used in epidemiological research, as illustrated by Demoury et al. [40], Armstrong-Hough et al. [47], Niedzwiedz et al. [48], Tang et al. [49], and Teng et al. [50], and Negative Binomial Regression fitted well in our models, accounting for the overdispersion issue, as also pointed out by Fairos et al. [18], while the Stone’s test is rarely used. The more recent studies we have found on this topic mainly deal with a small number of risk sources, such as Masseret et al. [39], Demoury et al. [40], Ha et al. [51], and Rodriguez-Villamizar et al. [52].

Our results also highlight that for SPs the relationship was stronger than for SBs. A possible reason may be that SPs are related to the hazard in emergence and development of *Ae*. *aegypti* larvae and mosquitoes. The spatial effect of SBs, associated to the risk to the exposure of dengue virus transmission, may be less pronounced as people infected in the vicinity may reside somewhere else in the city.

The integration of the Negative Binomial Regression and Stone’s test, show that SPs and SBs can play an important role in dengue transmission in Campinas. The use of both methods allowed us to show the risk depending on the distance from the sources.

Our results are coherent with other studies which found that these properties contribute to an increased risk of dengue transmission, especially the SP, establishing an important relationship between proximity to these places and the occurrence of dengue cases or incidence, like the studies conducted by Malavasi [11], Barbosa et al. [12], Johansen; do Carmo [13], and Mendes [14].

Barbosa et al. [15] and Santos et al. [16] however, did not find a relationship between SPs and an increased risk of dengue in their proximities. Barbosa et al. [15] also studied Campinas but considered only one year (from October 2015 to September 2016) and analyzed only four areas within the municipality. Santos et al. [16] studied Rio de Janeiro and considered the percentage of area occupied by these properties, but did not consider the distance from the SPs. In addition, data from Santos et al. [16] were aggregated by neighborhoods, a lower level of detail than used here. It is also important to mention that Rio de Janeiro has different historical, natural, social, economic, and cultural characteristics from those of Campinas.

It is important to recognize that in the present study, we did not account for the time spent in the exposed areas. Neither did we account for other environmental risk factors nor individual characteristics. Therefore, we recommend further investigation, in a more detailed approach, considering the aspects already mentioned and also considering not only the SPs/SBs as groups, but individualizing them—for example, according to their field of activity. Furthermore, to contribute to a better understanding of vector disease transmission dynamics, it would be important to add in a future model other variables associated to each SPs/SBs (such as the findings during the surveillance activities). Also recognizing the importance of these properties in the comprehension of dengue epidemics in Campinas and in other Brazilian municipalities, we suggest that in future analysis, it would be interesting to compare the SPs and SBs gradients with random location gradients.

We also highlight that although we have found that SPs and SBs can play an important role in dengue cases distribution in this municipality, other factors also may be associated with dengue, as already demonstrated by Marti et al. [53], Rocklöv; Tozan [54], Seidahmed et al. [55], and Whiteman et al. [56].

Additionally, we emphasize the importance of public agents’ survey work and the importance to keep and improve the inspections in SPs/SBs recorded in Campinas. Our analyses confirmed that these properties contribute to an increased number of dengue cases in their vicinity.

## Supporting information

S1 File Data supporting the statistical analyses conducted in this research article. The file contains information regarding the distance (meters) from putative risk sources (Strategic Points and Special Buildings), the population estimated for each range of distance, and the observed and expected number of dengue cases (2013–2016).
(PDF)

S2 File R Scripts used for statistical analyses in this research article. The file contains information regarding Poisson regression models, overdispersion tests, negative binomial regression models, likelihood ratio tests, Rate ratio and CI estimations, and Stone’s test.
(PDF)
