# Supplementary material for: Potential risk sites and their relationship with dengue cases, Campinas municipality, Southeast Brazil
Source: PLoS Negl Trop Dis. 2023 Apr 27;17(4):e0011237. doi: 10.1371/journal.pntd.0011237 (PMC10168557; doi:10.1371/journal.pntd.0011237)
Supplement: S1 File — The file contains information regarding the distance (meters) from putative risk sources (Strategic Points and Special Buildings), the population estimated for each range of distance, and the observed and expected number of dengue cases (2013–2016). (PDF) [file pntd.0011237.s001.pdf]

sp2013:

Dist_Mean	Observed	Pop
50	197	28644
150	365	63247
250	407	75775
350	558	117338
450	434	100015
550	421	108916
650	314	108019
750	292	100777
850	284	109198
950	234	101142

sp2013st:

Dist	Observed	Expected
0_100	197	110
100_200	365	243
200_300	407	291
300_400	558	451
400_500	434	384
500_600	421	418
600_700	314	415
700_800	292	387
800_900	284	419
900_1000	234	388

sp2014:

Dist_Mean	Observed	Pop
50	1596	29015
150	3880	79931
250	3984	92159
350	3739	105080
450	3148	107237
550	2939	113689
650	2505	116567
750	2427	117276
850	1932	90930
950	1891	96151

sp2014st:

Dist	Observed	Expected
0_100	1596	858
100_200	3880	2364
200_300	3984	2726
300_400	3739	3108
400_500	3148	3172
500_600	2939	3363
600_700	2505	3448
700_800	2427	3469
800_900	1932	2690
900_1000	1891	2844

sp2015:

Dist_Mean	Observed	Pop
50	2535	38271
150	5154	80330
250	7308	123707
350	6865	126096
450	6681	135773
550	5158	115995
650	4537	115592
750	3669	109696
850	2740	96172
950	2085	84588

sp2015st:

Dist	Observed	Expected
0_100	2535	1743
100_200	5154	3658
200_300	7308	5633
300_400	6865	5742
400_500	6681	6183
500_600	5158	5282
600_700	4537	5264
700_800	3669	4995
800_900	2740	4379
900_1000	2085	3852

sp2016:

Dist_Mean	Observed	Pop
50	131	26991
150	268	59745
250	364	79354
350	348	87983
450	323	89474
550	353	132952
650	273	118190
750	195	93234
850	203	98512
950	150	72983

sp2016st:

Dist	Observed	Expected
0_100	131	82
100_200	268	181
200_300	364	241
300_400	348	267
400_500	323	272
500_600	353	403
600_700	273	359
700_800	195	283
800_900	203	299
900_1000	150	221

sb2013:

Dist_Mean	Observed	Pop
50	27	5142
150	115	22099
250	171	38855
350	194	44343
450	214	52433
550	240	68986
650	232	71186
750	255	84684
850	206	74594
950	277	101482

sb2013st:

Dist	Observed	Expected
0_100	27	18
100_200	115	76
200_300	171	133
300_400	194	152
400_500	214	180
500_600	240	236
600_700	232	244
700_800	255	290
800_900	206	255
900_1000	277	348

sb2014:

Dist_Mean	Observed	Pop
50	206	5776
150	566	14349
250	1052	30526
350	1386	39151
450	1482	45994
550	1353	42127
650	1346	43743
750	1428	52418
850	1285	49244
950	1446	60512

sb2014st:

Dist	Observed	Expected
0_100	206	174
100_200	566	432
200_300	1052	919
300_400	1386	1178
400_500	1482	1384
500_600	1353	1268
600_700	1346	1316
700_800	1428	1577
800_900	1285	1482
900_1000	1446	1821

sb2015:

Dist_Mean	Observed	Pop
50	396	8618
150	1344	29672
250	1933	45691
350	1913	42076
450	2813	63949
550	2431	56553
650	2684	66759
750	2761	70856
850	3061	82557
950	2920	78968

sb2015st:

Dist	Observed	Expected
0_100	396	351
100_200	1344	1210
200_300	1933	1863
300_400	1913	1716
400_500	2813	2608
500_600	2431	2306
600_700	2684	2723
700_800	2761	2890
800_900	3061	3367
900_1000	2920	3221

sb2016:

Dist_Mean	Observed	Pop
50	26	7686
150	73	19653
250	193	56351
350	139	36753
450	205	61490
550	172	60687
650	129	40429
750	198	63502
850	167	52638
950	175	68805

sb2016st:

Dist	Observed	Expected
0_100	26	24
100_200	73	62
200_300	193	178
300_400	139	116
400_500	205	194
500_600	172	192
600_700	129	128
700_800	198	200
800_900	167	166
900_1000	175	217
