# Supplementary material for: Potential risk sites and their relationship with dengue cases, Campinas municipality, Southeast Brazil
Source: PLoS Negl Trop Dis. 2023 Apr 27;17(4):e0011237. doi: 10.1371/journal.pntd.0011237 (PMC10168557; doi:10.1371/journal.pntd.0011237)
Supplement: S2 File — The file contains information regarding Poisson regression models, overdispersion tests, negative binomial regression models, likelihood ratio tests, Rate ratio and CI estimations, and Stone’s test. (PDF) [file pntd.0011237.s002.pdf]

Scripts for the statistical analyses applied in the study “Potential risk sites and their relationship with dengue cases, Campinas municipality, Southeast Brazil”

STRATEGIC POINTS 2013*

First, import the file regarding the Strategic Points 2013 (sp2013)

Poisson Model

poissonmodel.sp2013=glm(Observed~Dist_Mean,offset=log(Pop),family= "poisson",data=sp2013)

summary(poissonmodel.sp2013)

Overdispersion Test

require(AER)

library (AER)

dispersiontest(poissonmodel.sp2013)

Negative Binomial Regression

install.packages("MASS")

library(MASS)

model.sp2013nb=glm.nb(Observed~Dist_Mean + offset(log(Pop)), data = sp2013)

summary(model.sp2013nb)

Perform a Likelihood Ratio Test to determine if there is a statistically significant difference in the fit of the two regression models:

pchisq(2 * (logLik(model.sp2013nb) - logLik(poissonmodel.sp2013)), df = 1, lower.tail = FALSE)

Rate Ratio and CI (Wald method)

install.packages ("epitools")

library (epitools)

rateratio.wald(c(3506,197,365,407,558,434,421,314,292,284,234, 913071,28644,63247,75775,117338,100015,108916,108019,100777,109198,101142))

The first number is the reference (total cases in the buffered area;); Next, the number of cases in each buffer size; Next, the population in the total buffered area; Finally, the populations in each buffer size.

Stone’s test

```
install.packages("spdep")
library(spdep)
install.packages("DCluster")
library (DCluster)
## Import the file regarding the Strategic Points 2013 prepared for the Stone’s test (sp2013st)
stone.test(Observed~offset(log(Expected)), sp2013st, model= "poisson", R=99, region=1, sorted=TRUE, lambda=1)
```

* The same procedure was applied to data regarding the Strategic Points 2014 (sp2014 and sp2014st), Strategic Points 2015 (sp2015 and sp2015st), Strategic Points 2016 (sp2016 and sp2016st), Special Buildings 2013 (sb2013 and sb2013st), Special Buildings 2014 (sb2014 and sb2014st), Special Buildings 2015 (sb2015 and sb2015st), and Special Buildings 2016 (sb2016 and sb2016st).
